# Ground Moving Target Tracking Filter Considering Terrain and Kinematics

**DOI:** 10.3390/s21206902

**Published:** 2021-10-18

**Authors:** Do-Un Kim, Woo-Cheol Lee, Han-Lim Choi, Joontae Park, Jihoon An, Wonjun Lee

**Affiliations:** 1 Department of Aerospace Engineering, Korea Advanced Institute of Science and Technology, Daejeon 34141, Korea; dukim@lics.kaist.ac.kr (D.-U.K.); wclee@lics.kaist.ac.kr (W.-C.L.); 2 LIG Nex1, Yongin-si 16911, Gyeonggi-do, Korea; joontae.park@lignex1.com (J.P.); jihoon.an@lignex1.com (J.A.); 3 Agency for Defense Development, Daejeon 34186, Korea; wonjun_lee@add.re.kr

**Keywords:** tracking filter, particle filter, soft constraint, DTED (digital terrain elevation model), gaussian process

## Abstract

This paper addresses ground target tracking (GTT) for airborne radar. Digital terrain elevation data (DTED) are widely used for GTT as prior information under the premise that ground targets are constrained on terrain. Existing works fuse DTED to a tracking filter in a way that adopts only the assumption that the position of the target is constrained on the terrain. However, by kinematics, it is natural that the velocity of the moving ground target is constrained as well. Furthermore, DTED provides neither continuous nor accurate measurement of terrain elevation. To overcome such limitations, we propose a novel soft terrain constraint and a constraint-aided particle filter. To resolve the difficulties in applying the DTED to the GTT, first, we reconstruct the ground-truth terrain elevation using a Gaussian process and treat DTED as a noisy observation of it. Then, terrain constraint is formulated as joint soft constraints of position and velocity. Finally, we derive a Soft Terrain Constrained Particle Filter (STC-PF) that propagates particles while approximately satisfying the terrain constraint in the prediction step. In the numerical simulations, STC-PF outperforms the Smoothly Constrained Kalman Filter (SCKF) in terms of tracking performance because SCKF can only incorporate hard constraints.

## 1. Introduction

Ground tracking radars mounted on airborne platforms play a key role in many applications, especially those for military purposes; surveillance, airstrike, and escort missions done by aircraft commonly require precise tracking of ground targets. In several modern military campaigns, ground moving target indicator (GMTI) radar on-board the Joint Surveillance Target Attack Radar System (STARS) has been proven strategically and tactically significant [1]. Accordingly, algorithms that track ground targets running on radars are becoming more important. Although there have been great advances in target tracking, tracking ground targets is still a challenging problem. The reason is that the characteristics of ground target tracking are different from those of tracking other types of targets. (e.g., high clutter, terrain obscuration, etc.) [2].

Because exploiting appropriate assumptions other than the state-space model can help to improve the statistical inferences of the system [3], many studies have tried to introduce useful assumptions to ground target tracking. They can be classified based on two criteria of what or how assumptions were applied.

Based on the first criteria, existing studies can be classified into two further categories. The first category considers the behavior characteristics of the ground target that are distinguished from those of airborne targets. Fosbury [4] and Kastella [5] each created the terms ‘trafficability’ and ‘hospitability of maneuver’, which represent how easily a vehicle can go through a particular area. These notions adaptively modify the target dynamics so that the dynamics can reflect the tendency of the target to prefer directions with small gradients. The second category involves empirical constraints. For instance, in the work of Streller [6], ground targets are assumed to move along the infrastructure such as roads, bridges, etc. More specifically, the assumption encourages the prior probability density which is propagated by the system model to align with the road network. The same assumption is utilized in the works of Pannetier [7,8]. The works of Mallick [9] and Kim [10] are also classified into the same category and share the same motivation as ours. To compensate for inaccurate GMTI measurements, both utilized the assumption that the position of a ground target is restricted to the terrain surface. This idea can be extended even further by adding another assumption that the velocity of the target is tangent to the terrain surface, which allowing the system to estimate the velocity more precisely [11,12].

From an other perspective, existing studies can be classified based on the second criteria, namely, how assumptions are applied. The first category involves modifying the target dynamics so that it can reflect the tendency of the target. Similar to the aforementioned works [4,5,6,13], the system dynamics of the filter are adaptively modified. In other words, external knowledge is embedded in the state-space model. Thus, we have the freedom to control only the tendency of a target. The second category involves transforming the assumption into a state constraint. This type of approach explicitly limits the state of a target to a specific subspace ([7,8] for example).

Extensive studies have attempted to deal with such constrained state estimation problems [11], including the methods that do not rely on the state-space model [14,15]. In the case of linear system dynamics and linear constraints, the following methods are applicable: model reduction [16], perfect measurement [17,18,19], estimate [20]/system [21]/gain [22] projection, pdf truncation [22], etc. If either system dynamics or constraint is nonlinear, the combination of linearization and linear methods is an available option. Other possible choices are variants of the Unscented Kalman Filter (PUKF [12,23], ECUKF [12], 2UKF [24], etc.), variants of the Particle Filter [25,26,27,28] (CLIP, COMP [29]), and the Smoothly Constrained Kalman Filter (SCKF) [30]. Moreover, many works in the literature have paid attention to state estimation problems with soft constraints [18,19,31,32,33,34]. Soft constraints, conditions that the state approximately satisfies, are utilized in most practical engineering applications [11,33] because uncertainty may appear during the transformation of external knowledge into the constraint. For example, in the case of ground target tracking constrained to a road, the roadmap may be inaccurate. Among promising methods dealing with soft constraints, some regard the degree of constraint satisfaction as measurement and extend the likelihood function [18,19,31,32,35]. Especially, this approach can be intuitively extended to a nonlinear soft constraint; scPF (soft-constrained Particle Filter) [35] is a good example. scPF has the advantage of preserving the nonlinearity of the constraint because it is based on an SIR (Sequential Importance Resampling) particle filter. However, scPF is not sample-efficient because the constraint is reflected by the generalized likelihood. More specifically, while particles are propagated through the system dynamics, they can be scattered in a direction that does not satisfy the constraint. Therefore, the propagated particles that do not satisfy the constraint would be given a low likelihood and eventually vanish, which makes the whole algorithm inefficient.

Thus, in this paper, we propose a particle filter that considers the stochastic terrain constraint. The term ‘terrain constraint’ not only represents the assumption that the position of a ground target should be located on the terrain surface but also that the velocity vector of the target should be tangent to the terrain surface. Contributions are the following:

- **We propose a sample-efficient particle filter to which the terrain constraint can be applied.** The proposed algorithm is named Soft Terrain Constrained Particle Filter (STC-PF). Given the assumption of target motion, STC-PF performs sampling in a direction for which the state satisfies the constraint during the propagation step. As a result, STC-PF is more sample-efficient than scPF. Furthermore, in the numerical simulations, STC-PF using soft terrain constraint outperforms Smoothly Constrained Kalman Filter (SCKF) [30] using hard constraint in terms of tracking performance.
- **Using a Gaussian process, terrain constraint is formulated as a soft position constraint along with a soft velocity constraint.** Because kinematics states that position and velocity is not independent, a constraint on the position of a target implies that the velocity of the target will be constrained as well. Therefore, terrain constraint includes both position constraint and velocity constraint. Furthermore, terrain constraint requires exact terrain elevation and its gradient at an arbitrary position, but DTED (Digital Terrain Elevation Data) [36] cannot provide them. To overcome this issue, we model the ground-truth terrain elevation with a Gaussian process (GP) and treat DTED as a noisy observation [37] of it.

Technically, we used SRTM (Shuttle Radar Topography Mission). However, we will use the term DTED and SRTM interchangeably as they both are data that map terrain elevation of the entire globe.

The structure of this paper is as follows: In Section 2, tracking of a ground target with a terrain constraint is formulated. Section 3 presents the proposed algorithm, STC-PF. Section 4 provides detailed explanations, the results, and a discussion of the numerical simulation. Finally, in Section 5, we conclude.

## 2. Problem Formulation

In this section, tracking of a ground target with terrain constraint is formulated as a constrained state estimation problem.

Consider a system described by the following state-space model:

(1) $$x_{k+1}=f\left(x_{k}\right)+w_{k}$$

(2) $$y_{k}=g\left(x_{k}\right)+n_{k}$$

where $x_{k}$ is the system state vector at time *k*, $y_{k}$ the measurement vector, f the system function, g the observation function, $w_{k}$ the process noise vector, and $n_{k}$ the measurement noise vector. The system state vector $x_{k}\in R^{6}$ consists of the position ($x_{k}$, $y_{k}$, $z_{k}$) and the velocity ($v_{x,k}$, $v_{y,k}$, $v_{z,k}$) in local Cartesian coordinates at time *k*. The system function is a possibly nonlinear function but is assumed to be a constant velocity model in this paper. $y_{k}\in R^{3}$ is the measurement, which consists of range, azimuth angle, and elevation angle measured from the radar. $w_{k}∼N\left(0,Q\right)$ is white Gaussian process noise, and $n_{k}∼N\left(0,R\right)$ is white Gaussian measurement noise. Subsequently, Equations (1) and (2) are realized as follows:

(3) $$x_{k+1}=\left[\begin{array}{cc} I_{3\times 3} & \Delta t\cdot I_{3\times 3}\\ 0_{3\times 3} & I_{3\times 3} \end{array}\right]x_{k}+w_{k}$$

(4) $$y_{k}=\left[\begin{array}{c} \sqrt{x_{k}^{2}+y_{k}^{2}+z_{k}^{2}}\\ \arctan\frac{y_{k}}{x_{k}}\\ \arcsin\frac{z_{k}}{\sqrt{x_{k}^{2}+y_{k}^{2}+z_{k}^{2}}} \end{array}\right]+n_{k}.$$

The final goal of the state estimation problem is to infer the state sequence of the dynamical system $x_{0:k}$ from the series of observations $y_{1:k}$.

Now, the terrain constraint can come into play to incorporate the additional information that the state-space model cannot reflect. The terrain constraint not only represents the assumption that the position of a ground target should be located on the terrain surface but also that the velocity vector of the target should be tangent to the terrain surface. Both assumptions can be transformed into state constraints as follows:

(5) $$\begin{array}{c} h_{k}=\bar{h}\left(\lambda_{k},\varphi_{k}\right)\\ v_{h,k}=\nabla \bar{h}\left(\lambda_{k},\varphi_{k}\right)\cdot {\left[\begin{array}{cc} v_{\lambda ,k} & v_{\varphi ,k} \end{array}\right]}^{T} \end{array}$$

where $\lambda_{k}$, $\varphi_{k}$, and $h_{k}$ are the latitude, longitude, and altitude (LLA) of the target at time *k*. $\bar{h}\left(\lambda ,\varphi \right)$ is ground-truth terrain elevation at latitude λ and longitude φ. Note that we do not have direct access to $\bar{h}$, but only noisy observations,

(6) $$D=\{\mathrm{DTED}\left(\lambda^{i},\varphi^{i}\right)|i=1\cdots N_{D}\}$$

such that

(7) $$\mathrm{DTED}\left(\lambda ,\varphi \right)=\bar{h}\left(\lambda ,\varphi \right)+\epsilon \left(\lambda ,\varphi \right).$$

## 3. Soft Terrain Constrained Particle Filter

In this section, the newly proposed algorithm, Soft Terrain Constrained Particle Filter (STC-PF) is derived. In Section 3.1, mathematical modeling of ground-truth terrain elevation is presented. Then, we propose a technique for the transformation of velocity between the LLA coordinates and the local Cartesian coordinates in Section 3.2. Necessary assumptions required for algorithm derivation are described in Section 3.3. After the algorithm derivation in Section 3.4, we show the similarity between STC-PF and scPF [35] in Section 3.5.

### 3.1. Modeling of Ground-Truth Terrain Elevation

**Figure 1:**
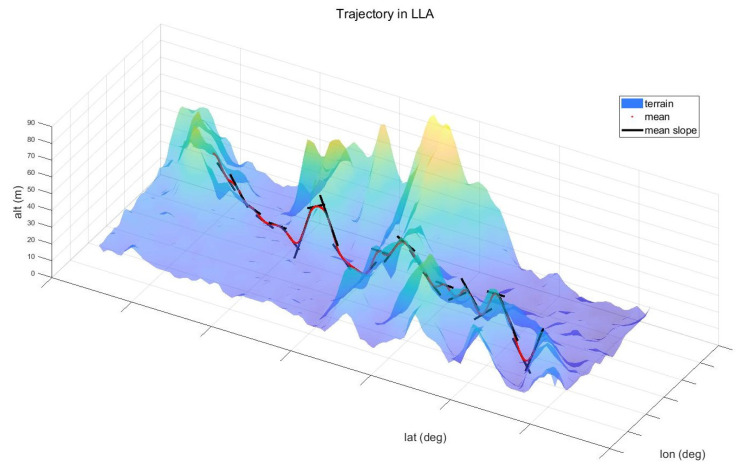
GP Prediction Example.

Although the terrain constraint (Equation (5)) requires the ground-truth elevation, it is almost impossible in practice to retrieve it at an arbitrary position. The reason is that DTED provides neither accurate ground-truth terrain elevation (Equation (7)) nor terrain elevation at arbitrary positions. (Equation (6)) This challenge can be met by reconstructing the ground-truth terrain elevation with a Gaussian process (GP) and treating the DTED as independent observations:

(8) $$\begin{array}{c} \bar{h}∼GP\left(m\left(\lambda ,\varphi \right),k(\left(\lambda ,\varphi \right),\left(\lambda^{\prime },\varphi^{\prime }\right))\right)\\ \mathrm{DTED}\left(\lambda ,\varphi \right)=\bar{h}\left(\lambda ,\varphi \right)+\epsilon \\ \epsilon ∼N(0,\sigma_{\mathrm{DTED}}) \end{array}$$

where the observation noise $\sigma_{\mathrm{DTED}}$ can be estimated from the work of Rodriguez [37]. (see Appendix B) Because GP assigns a probability for each possible terrain, the terrain constraint becomes stochastic. An advantage of this approach is that it enables us to compute the gradient of $\bar{h}$ analytically, which is required to apply the velocity constraint. (Equation (5)) More strictly, joint predictive distribution for ground-truth terrain elevation and its gradient can be expressed in a closed-form, (detailed description is in Appendix A)

(9) $$\bar{h},\nabla \bar{h}\;|\;\mathrm{DTED}∼N\left(\bar{\mu },\bar{\Sigma }\right)$$

provided that the kernel function is differentiable. Figure 1 shows an example of prediction results when zero mean function and squared exponential kernel are utilized.

(10) $$\begin{array}{cc} m(\lambda ,\varphi ) & =E\left[\bar{h}\left(\lambda ,\varphi \right)\right]\\ & =0\\ k\left(\left(\lambda ,\varphi \right),\left(\lambda^{\prime },\varphi^{\prime }\right)\right) & =E\left[\left(\bar{h}\left(\lambda ,\varphi \right)-m\left(\lambda ,\varphi \right)\right)\left(\bar{h}\left(\lambda^{\prime },\varphi^{\prime }\right)-m\left(\lambda^{\prime },\varphi^{\prime }\right)\right)\right]\\ & =\alpha \exp\left(-\frac{1}{2}\left[\begin{array}{cc} \lambda -\lambda^{\prime } & \varphi -\varphi^{\prime } \end{array}\right]\Gamma \left[\begin{array}{c} \lambda -\lambda^{\prime }\\ \varphi -\varphi^{\prime } \end{array}\right]\right) \end{array}$$

### 3.2. Velocity Transformation

Another major challenge when applying the terrain constraint to the filter is that the conversion of velocity between the local Cartesian coordinates and the LLA coordinates is not straightforward. More specifically, the terrain constraint (Equation (5)) requires the velocity in LLA coordinates.

This challenge can be met by multiplying the Jacobian, which is obtained by numerical differentiation. Additionally, because velocity in local Cartesian coordinates is relative while that in LLA coordinates is absolute, the velocity of the radar $V_{lla,ownship}$ should be added (or subtracted) after (or before) multiplying by the Jacobian.

(11) $$\begin{array}{cc} {\left[\begin{array}{c} \frac{\partial \lambda }{\partial t}\\ \frac{\partial \varphi }{\partial t}\\ \frac{\partial h}{\partial t} \end{array}\right]}_{rel}\; & =\left[\begin{array}{ccc} \frac{\partial \lambda }{\partial x} & \frac{\partial \lambda }{\partial y} & \frac{\partial \lambda }{\partial z}\\ \frac{\partial \varphi }{\partial x} & \frac{\partial \varphi }{\partial y} & \frac{\partial \varphi }{\partial z}\\ \frac{\partial h}{\partial x} & \frac{\partial h}{\partial y} & \frac{\partial h}{\partial z} \end{array}\right]\left[\begin{array}{c} \frac{\partial x}{\partial t}\\ \frac{\partial y}{\partial t}\\ \frac{\partial z}{\partial t} \end{array}\right]\\ \; & =J_{xyz2lla}V_{xyz}\\ V_{lla} & =J_{xyz2lla}V_{xyz}+V_{lla,ownship} \end{array}$$

Conversion from LLA to local Cartesian can be done in a converse way.

(12) $$V_{xyz}=J_{lla2xyz}\left(V_{lla}-V_{lla,ownship}\right)$$

where $J_{lla2xyz}=J_{xyz2lla}^{-1}$.

### 3.3. Assumptions

Regarding the motion of the target, we assume the followings:

1. The vertical position (*h*) can be determined provided that the horizontal position (λ, φ) is fixed.
2. Then, the vertical velocity ($v_{h}$) can be also determined when the horizontal velocity ($v_{\lambda }$, $v_{\varphi }$) is fixed.

**Figure 2:**
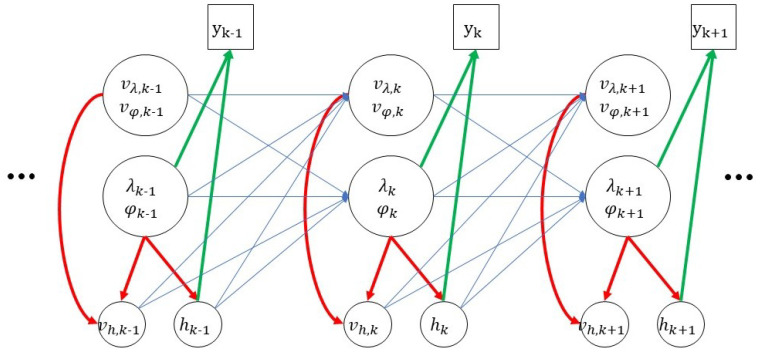
Bayesian Network Representation of Target Motion.

In Figure 2, assumptions 1 and 2 correspond to the red arrows that inbound to *h* and $v_{h}$, respectively. They comprise the ’elevation model’.

Due to the recursive Markovian structure, it is possible to infer the current latent state from the previously inferred latent state and the current measurement. Mathematically, by Bayes’ rule, the joint distribution of $x_{0:k}$ given $y_{1:k}$ can be expressed as

(13) P(x0:k|y1:k)∝·P(yk|λk,φk,hk)·P(λk,φk,vλ,k,vφ,k|xk−1)·P(hk|λk,φk)·P(vh,k|λk,φk,vλ,k,vφ,k)·P(x0:k−1|y0:k−1)

The dynamic model $P(\lambda_{k},\varphi_{k},v_{\lambda ,k},v_{\varphi ,k}|x_{k-1})$ and the likelihood model $P(y_{k}|\lambda_{k},\varphi_{k},h_{k})$ are found in the above equation. Respectively, they correspond to the blue arrows and the green arrows in Figure 2. Note that the measurement $y_{k}$ is only affected by the position of the target ($\lambda_{k}$, $\varphi_{k}$, $h_{k}$), as stated in Equation (4).

### 3.4. Algorithm

The proposed algorithm is based on the SIR(Sequential Importance Resampling) particle filter. In the SIR algorithm, which forms the basis of most sequential Monte Carlo (MC) filters [38], the posterior probability density function $P(x_{0:k}|y_{1:k})$ is characterized by the set of support points ${\left\{x_{0:k}^{i}\right\}}_{i=1}^{N_{p}}$ (or particles) and the corresponding weights ${\left\{w_{k}^{i}\right\}}_{i=1}^{N_{p}}$, where $N_{p}$ is the number of particles [39]. The posterior density at time *k* is approximated as

(14) $$P\left(x_{0:k}|y_{1:k}\right)\approx \sum_{i=1}^{N_{p}}w_{k}^{i}\delta \left(x_{0:k}-x_{0:k}^{i}\right)$$

such that

(15) $$\sum_{i=1}^{N_{p}}w_{k}^{i}=1,$$

where δ(·) represents the Dirac delta function. We assume that the particles are sampled from a well-known proposal distribution,

(16) $$x_{0:k}^{i}∼q\left(x_{0:k}^{i}|y_{1:k}\right).$$

Then, by the principle of importance sampling, the corresponding weight is calculated as

(17) $$w_{k}^{i}\propto \frac{P(x_{0:k}^{i}|y_{1:k})}{q(x_{0:k}^{i}|y_{1:k})}.$$

Because we have freedom to choose the proposal distribution, we consider a proposal distribution that has a form of

(18) $$q\left(x_{0:k}|y_{1:k}\right)=q\left(x_{k}|x_{0:k-1},y_{1:k}\right)q\left(x_{0:k-1}|y_{1:k-1}\right).$$

In other words, one can draw new support points $x_{0:k}^{i}∼q\left(x_{0:k}|y_{1:k}\right)$ by augmenting each of the previous support points $x_{0:k-1}^{i}∼q\left(x_{0:k-1}|y_{1:k-1}\right)$ with the new state $x_{k}^{i}∼q\left(x_{k}|x_{0:k-1},y_{1:k}\right)$.

Starting from Equation (17),

(19) $$w_{k}^{i}\propto w_{k-1}^{i}\frac{P(x_{0:k}^{i}|y_{1:k})}{P(x_{0:k-1}^{i}|y_{1:k-1})}\frac{q(x_{0:k-1}^{i}|y_{1:k-1})}{q(x_{0:k}^{i}|y_{1:k})}.$$

Together with Equation (18) and the recursive relation (Equation (13)), the weight update equation can be simplified.

(20) $$w_{k}^{i}\propto w_{k-1}^{i}\frac{P\left(y_{k}|\lambda_{k}^{i},\varphi_{k}^{i},h_{k}^{i}\right)\cdot P\left(\lambda_{k}^{i},\varphi_{k}^{i},v_{\lambda ,k}^{i},v_{\varphi ,k}^{i}|x_{k-1}^{i}\right)\cdot P\left(h_{k}^{i}|\lambda_{k}^{i},\varphi_{k}^{i}\right)\cdot P\left(v_{h,k}^{i}|\lambda_{k}^{i},\varphi_{k}^{i},v_{\lambda ,k}^{i},v_{\varphi ,k}^{i}\right)}{q(x_{k}^{i}|x_{0:k-1}^{i},y_{1:k})}$$

A further assumption regarding the proposal distribution,

(21) $$q\left(x_{k}^{i}|x_{0:k-1}^{i},y_{1:k}\right)=P\left(\lambda_{k}^{i},\varphi_{k}^{i},v_{\lambda ,k}^{i},v_{\varphi ,k}^{i}|x_{k-1}^{i}\right)\cdot P\left(h_{k}^{i}|\lambda_{k}^{i},\varphi_{k}^{i}\right)\cdot P\left(v_{h,k}^{i}|\lambda_{k}^{i},\varphi_{k}^{i},v_{\lambda ,k}^{i},v_{\varphi ,k}^{i}\right)$$

yields

(22) $$w_{k}^{i}\propto w_{k-1}^{i}\cdot P\left(y_{k}|\lambda_{k}^{i},\varphi_{k}^{i},h_{k}^{i}\right)$$

This means that the weight of each particle is updated proportionally to its corresponding likelihood. Note that the above weight update equation implicitly includes the normalization given by Equation (15).

The proposed algorithm, STC-PF, is summarized in Algorithm 1. In a vanilla SIR PF, the next state is propagated through the dynamic model only. In contrast, in STC-PF, the next state is propagated through the dynamic model first and then propagated through the elevation model (line numbers 1 and 1 in Algorithm 1).

**Figure 3:**
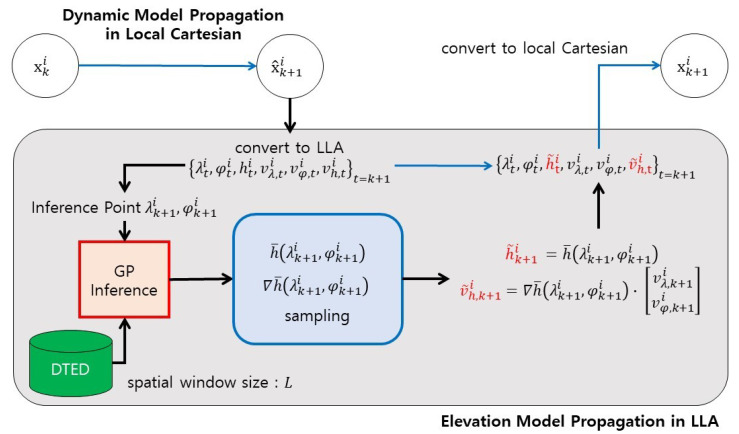
Implementation of Elevation Model Propagation.

In Figure 3, a detailed implementation of the elevation model propagation is shown. It is worth mentioning that elevation model propagation can be accelerated by two techniques: parallelization and use of local data during the GP inference. Because the propagation process for each particle does not require information on other particles, it can be parallelized. Furthermore, during the GP inference, only the neighborhood data of DTED are utilized. The range of the neighborhood is defined by the spatial window size *L*.
**Algorithm 1:** Soft Terrain Constrained Particle Filter (STC-PF).
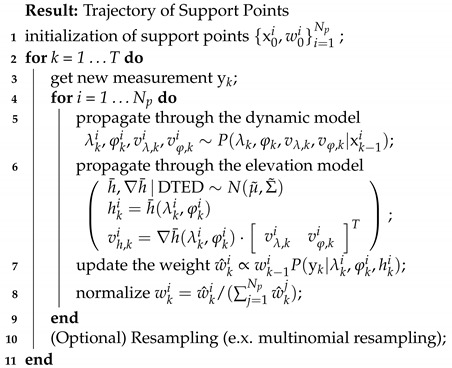


### 3.5. Remark on an Existing Work

As mentioned in Section 1, from a mathematical perspective, the proposed algorithm (STC-PF) is similar to scPF (soft-constrained Particle Filter) [35]. Similar to STC-PF, scPF is based on the SIR particle filter; however, the two differ in the sense that scPF utilizes generalized likelihood.

(23) $${\hat{w}}_{k}^{i}\propto w_{k-1}^{i}P\left(y_{k}|x_{k}^{i}\right)P\left(C_{k}|x_{k}^{i}\right)$$

where $P(C_{k}|x_{k}^{i})$ is a pseudo-measurement that represents how much the given state $x_{k}^{i}$ satisfies the constraint. If Equation (21) is replaced by

(24) $$q\left(x_{k}^{i}|x_{0:k-1}^{i},y_{1:k}^{i}\right)=P\left(\lambda_{k}^{i},\varphi_{k}^{i},v_{\lambda ,k}^{i},v_{\varphi ,k}^{i}|x_{k-1}^{i}\right),$$

then the weight update rule is also changed.

(25) $$w_{k}^{i}\propto w_{k-1}^{i}\cdot P\left(y_{k}|\lambda_{k}^{i},\varphi_{k}^{i},h_{k}^{i}\right)\cdot P\left(h_{k}^{i}|\lambda_{k}^{i},\varphi_{k}^{i}\right)\cdot P\left(v_{h,k}^{i}|\lambda_{k}^{i},\varphi_{k}^{i},v_{\lambda ,k}^{i},v_{\varphi ,k}^{i}\right)$$

Thus, the generalized likelihood function can be identified by equating the elevation model with the pseudo-measurement. As a result, scPF can be reduced to STC-PF as long as the assumption for target motion holds.

## 4. Simulation

### 4.1. Scenario and Parameter Settings

**Figure 4:**
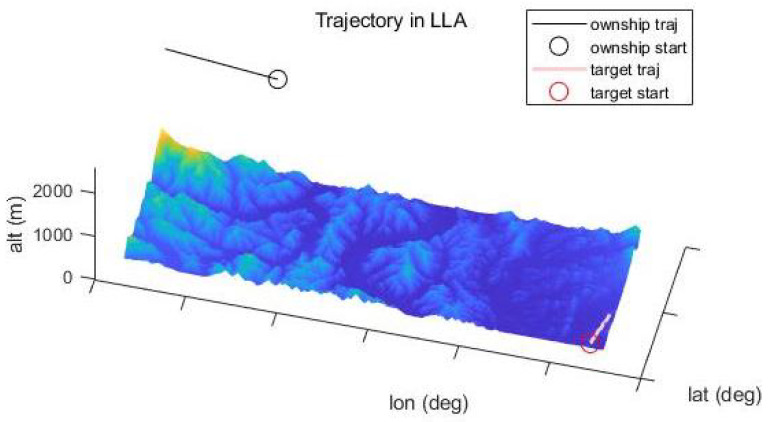
Trajectory in WGS84 LLA (0.05 degree interval).

To evaluate STC-PF, numerical experiments are performed with the following scenario: The radar is mounted on an aircraft that flies at a speed of 70 m/s at a height of 2500 m. The radar tracks a single target that moves along the surface at a speed of 25 m/s. (see Figure 4) The simulation runs for 100 s. Furthermore, to reflect the uncertainty in DTED, a noisy version of DTED is created. More specifically, iid zero-mean Gaussian noise with variance $\sigma_{\mathrm{DTED}}$ is sampled and added for each data entry in DTED. Because it is reasonable to bound the uncertainty of DTED, sampled noise is clipped to 50 m if its absolute value exceeds 50 m.

Values of parameters used in the simulation are listed in Table 1. Detailed explanation about the choice of GP hyper-parameters is in the Appendix B. The simulations are performed with two settings that differ in the value of $\sigma_{\mathrm{DTED}}$. The reasonable value for $\sigma_{\mathrm{DTED}}$ is 3.77 m, which is inferred from [37]. However, another setting whose $\sigma_{\mathrm{DTED}}$ is 1.89 m is also used to observe the sensitivity of the key parameter.

### 4.2. Baseline Methods

To compare STC-PF with other filters that can incorporate nonlinear constraints, the Smoothly Constrained Kalman Filter (SCKF) is implemented as well [30]. Note that ‘Smoothly Constrained’ in the name of SCKF does not mean soft constraint. Because SCKF can incorporate only deterministic constraints, it requires approximations of ground-truth terrain elevation that require *h* and ∇h to be fixed to specific values. One approach used for the comparison is to ignore the noise inherent in DTED and use bilinear interpolation to retrieve the terrain elevation at arbitrary positions.

(26) $$\begin{array}{c} E\left(x_{k}\right)=h_{k}-\bar{h}\left(\lambda_{k},\varphi_{k}\right)=0\\ E_{v}\left(x_{k}\right)=v_{h,k}-\nabla \bar{h}\left(\lambda_{k},\varphi_{k}\right)\cdot {\left[\begin{array}{cc} v_{\lambda ,k} & v_{\varphi ,k} \end{array}\right]}^{T}=0\\ \bar{h}\left(\lambda ,\varphi \right)\approx BL\left(\lambda ,\varphi \;|\;\mathrm{DTED}\right) \end{array}$$

where BL(λ,φ|DTED) is bilinear interpolation at (λ,φ) given DTED. For the computation of the gradient $\nabla \bar{h}$, central numerical differentiation is used instead of analytic differentiation to avoid non-differentiable cases.

(27) $${\left.\frac{\partial \bar{h}}{\partial \lambda }\right|}_{x_{k}}\approx \frac{\bar{h}\left(\lambda_{k}+\Delta ,\varphi_{k}\right)-\bar{h}\left(\lambda_{k}-\Delta ,\varphi_{k}\right)}{2\Delta }$$

where Δ is a small constant.

Another method is to use GP mean regression rather than bilinear interpolation. That is,

(28) $${\left[\begin{array}{cc} \bar{h} & \nabla \bar{h} \end{array}\right]}^{T}\approx \bar{\mu }$$

where $\bar{\mu }$ is the GP joint mean of $\bar{h}$ and $\nabla \bar{h}$ in Equation (9). This enables us to reconstruct the most probable ground-truth terrain elevation considering the noise of DTED; however, this method still cannot consider the uncertainty of the inferred $\bar{h}$ and $\nabla \bar{h}$ values, in contrast to STC-PF.

SCKF requires the Jacobian of the constraint functions:

(29) $$\begin{array}{ccc} G(x_{k})= & {\left.\frac{\partial E}{\partial x}\right|}_{x_{k}}= & {\left.\left[\begin{array}{cccccc} \frac{\partial E}{\partial x} & \frac{\partial E}{\partial y} & \frac{\partial E}{\partial z} & \frac{\partial E}{\partial v_{x}} & \frac{\partial E}{\partial v_{y}} & \frac{\partial E}{\partial v_{z}} \end{array}\right]\right|}_{x_{k}}\\ G_{v}\left(x_{k}\right)= & {\left.\frac{\partial E_{v}}{\partial x}\right|}_{x_{k}}= & {\left.\left[\begin{array}{cccccc} \frac{\partial E_{v}}{\partial x} & \frac{\partial E_{v}}{\partial y} & \frac{\partial E_{v}}{\partial z} & \frac{\partial E_{v}}{\partial v_{x}} & \frac{\partial E_{v}}{\partial v_{y}} & \frac{\partial E_{v}}{\partial v_{z}} \end{array}\right]\right|}_{x_{k}} \end{array}$$

However, it is impossible to differentiate $E(x_{k})$ and $E_{v}\left(x_{k}\right)$ analytically because they involve coordinate transformation between local Cartesian and WGS84 LLA. Alternatively, the derivative can be obtained using the central numerical difference regardless of the regression method.

(30) $${\left.\frac{\partial E}{\partial x}\right|}_{x_{k}}\approx \frac{E\left(x_{k}+\Delta \cdot e_{x}\right)-E\left(x_{k}-\Delta \cdot e_{x}\right)}{2\Delta },$$

where $e_{x}$ is a canonical unit vector whose first component is nonzero. $\partial E/\partial y_{k}$, $\partial E/\partial z_{k}$, and $\partial E_{v}/\partial \;\circ$ can be obtained in a similar way. Because *E* is not a function of $v_{\cdot ,k}$, corresponding derivatives automatically become zero.

### 4.3. Results

To evaluate STC-PF, SCKF using bilinear regression, and SCKF using GP mean regression, 100 Monte-Carlo simulations were carried out for each $\sigma_{\mathrm{DTED}}$ value. Tracking performance is assessed based on timewise RMS (Root Mean Squared) error. For example, timewise RMS for local Cartesian *x* position error at time *k* is

(31) $$RMS_{x,k}=\sqrt{\frac{1}{N_{MC}}\sum_{n=1}^{N_{MC}}{\left(x_{k}^{n}-{\bar{x}}_{k}\right)}^{2}}$$

where $N_{MC}$ is the number of repetitions (i.e., 100), $x_{k}^{n}$ the filter mean value for *x* position at time *k* in the $n^{th}$ trial, and ${\bar{x}}_{k}$ the ground-truth *x* position at time *k*. The time average (10≤k≤90) for timewise RMS is also computed for evaluation.

**Figure 5:**
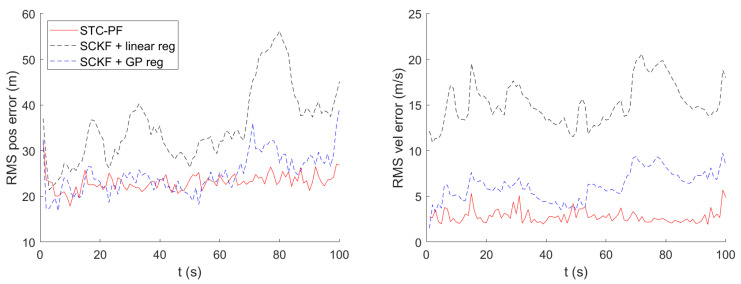
Timewise RMS for Local Cartesian Position and Velocity Error ($\sigma_{\mathrm{DTED}}=3.77$ m).

**Figure 6:**
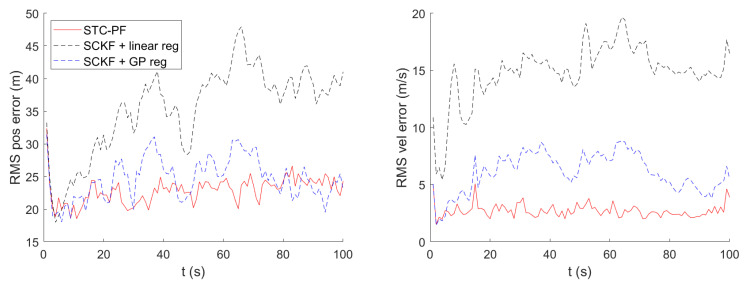
Timewise RMS for Local Cartesian Position and Velocity Error ($\sigma_{\mathrm{DTED}}=1.89$ m).

Figure 5 shows the timewise RMS for local Cartesian position error and velocity error. In the figures, SCKF using bilinear regression shows the worst tracking performance. In terms of time average of RMS position error, as shown in Table 2, the superiority of STC-PF over SCKF using GP mean regression is clear, although it cannot be identified in Figure 5. In terms of RMS velocity error, STC-PF distinctly outperforms the other two methods. This trend also holds for the different parameter setting, namely $\sigma_{\mathrm{DTED}}$ = 1.89 m, as shown in Figure 6 and Table 3.

On the other hand, the speed of the algorithms is assessed based on the average processing time for a single timestep. STC-PF and SCKF both were implemented in MATLAB and run on an Intel Core i7-8565U with 16.0GB RAM. Table 4 shows that the baseline method runs nearly in real time, while STC-PF does not. Nevertheless, with parallel computing, STC-PF gets much faster and shows the possibility of real time applications.

### 4.4. Discussion

According to the simulation results, the tracking performance of SCKF depends on the regression method. More specifically, SCKF using GP mean regression has smaller RMS errors than SCKF using bilinear regression. This result could be due to the regression method used for target trajectory generation: GP regression, which is suitable for fitting of a smooth curve, might result in a trajectory closer to the ground-truth trajectory generated by bicubic spline interpolation.

Meanwhile, the superiority of STC-PF over SCKF in terms of tracking performance could stem from two factors. The first is that a particle filter is more expressive than a simple Gaussian filter. Because SCKF assumes that the posterior distribution is a simple Gaussian, SCKF adjusts its state to meet the constraint at one point. However, unlike SCKF, particle filters do not assume the form of a posterior distribution. Therefore, STC-PF can estimate the distribution of the velocity hypothesis for each position hypothesis, so that the combined hypothesis independently meets the terrain constraint. As a result, the filter mean value, a weighted sum of each hypothesis, is less biased.

**Figure 7:**
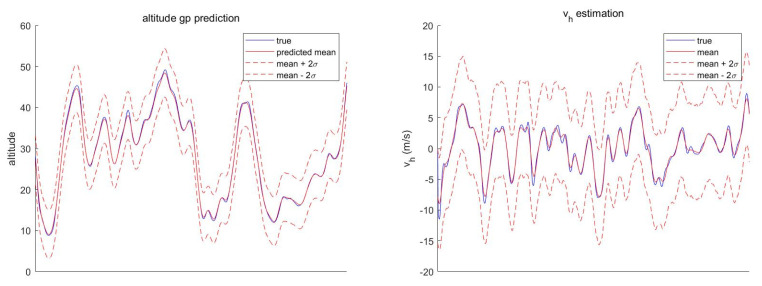
GP Prediction Result Without Position Uncertainty and DTED Noise.

**Figure 8:**
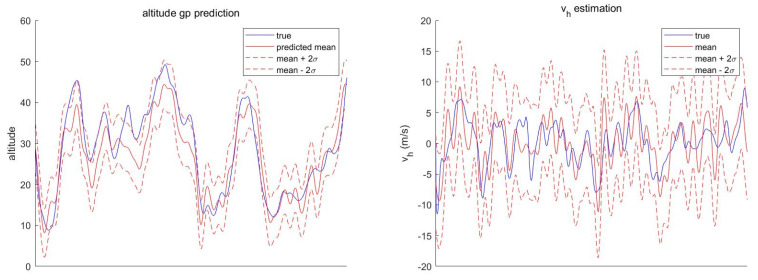
GP Prediction Result with both Position Uncertainty and DTED Noise.

The second reason is that the state estimation with soft constraint is less sensitive to the uncertainty of the constraint. Conversely, state estimation with hard constraint is very sensitive to the uncertainty of the constraint. Figure 7 shows that the terrain constraint (Equation (5)) holds almost perfectly assuming perfect knowledge of the position and DTED. However, the hard constraint, the GP mean regression, deviates from the ground-truth value if small amounts of position uncertainty (approximately 30 m in the longitudinal direction) and DTED noise ($\sigma_{\mathrm{DTED}}=3.77$ m) are introduced (see Figure 8). In other words, uncertainty of the horizontal position and DTED can result in a catastrophic state error if a hard constraint is applied. On the other hand, STC-PF can absorb the error to some degree as most of the ground-truth value resides inside the two-sigma bound.

Regarding the speed of the algorithm, it is natural that STC-PF consumes more computational resources than SCKF. This is because STC-PF requires expensive computation for every particle; GP inference whose time complexity is $O(n^{3})$, where *n* is the number of observation points involved in the GP inference. Fortunately, time consumption is drastically reduced by the virtue of parallel computing, and it can be reduced further by two measures. One is reducing the number of GP observation points (namely, the spatial window *L* in Figure 3) as far as the performance degradation is negligible, and the other is increasing the number of cores dedicated to the filtering.

## 5. Conclusions

To sum up, we have proposed a particle filter to improve the performance of ground target tracking. To estimate the velocity more accurately, not only a position constraint but also a velocity constraint has been introduced in the terrain constraint. Although DTED provides terrain elevation of the entire globe, it provides inaccurate values at discrete positions. Thus, the ground-truth terrain elevation included in the terrain constraint has been modeled with a Gaussian process, and DTED has been regarded as noisy observations of it. As a result, terrain constraint has become a soft constraint that can reflect the uncertainty of DTED. Finally, we have proposed a particle filter, STC-PF, given the assumption of the motion of the target. STC-PF is based on SIR PF, but the major difference is that STC-PF uses the elevation model. Due to the elevation model, knowledge of the horizontal position and velocity of a target enables us to infer the vertical position and velocity more precisely. In the numerical simulation, STC-PF has been compared with SCKF which can incorporate hard constraints only. Furthermore, to reflect the uncertainty in DTED, filters have made use of DTED contaminated by noise, whereas the ground-truth trajectory of the target is generated by the original DTED. The simulation results showed that STC-PF outperforms SCKF in terms of RMS error, for two possible reasons. The first is that particle filters are more expressive than simple Gaussian filters. The second is that the state estimation with soft constraint is less sensitive to uncertainty of the constraint than that with hard constraint.

## Figures and Tables

**Table 1:** Parameter Setting.

Name	Value
$\sigma_{\mathrm{DTED}}\;\left(m\right)$	3.77, 1.89
α	100
$\Gamma \;(deg^{-2})$	$\mathrm{diag}\left[\begin{array}{cc} \frac{1}{{\left(2.78e-4\right)}^{2}} & \frac{1}{{\left(2.78e-4\right)}^{2}} \end{array}\right]$
L(arcsec)	13(≈390m)
Δt (s)	1.0
Initial Cov.	$\left[\begin{array}{cc} 1e2\left(m^{2}\right)\cdot I_{3\times 3} & 0_{3\times 3}\\ 0_{3\times 3} & 10\left(m^{2}/s^{2}\right)\cdot I_{3\times 3} \end{array}\right]$
$N_{p}$	1e4
*Q*	20(m)·I3×3003×3002(m/s)0003×302(m/s)00005(m/s)2
*R*	${\left(\mathrm{diag}\left[\begin{array}{ccc} 10(m) & 0.1(deg) & 0.1(deg) \end{array}\right]\right)}^{2}$

**Table 2:** Time Average of Timewise RMS ($\sigma_{\mathrm{DTED}}=3.77$ m).

		SCKF	SCKF
	STC-PF	+	+
		Bilinear	GP
x(m)	9.61	10.9	9.52
*y*	20.7	34.1	22.4
*z*	2.77	3.84	3.05
Position	23.0	36.1	24.6
$v_{x}\;$(m/s)	0.972	4.10	1.55
$v_{y}$	1.74	14.0	5.45
$v_{z}$	1.78	4.16	2.15
Velocity	2.75	15.4	6.15

**Table 3:** Time Average of Timewise RMS ($\sigma_{\mathrm{DTED}}=1.89$ m).

		SCKF	SCKF
	STC-PF	+	+
		Bilinear	GP
*x* (m)	9.48	11.0	9.63
*y*	20.5	34.4	23.1
*z*	2.56	3.96	3.12
Position	22.8	36.4	25.3
$v_{x}$(m/s)	0.966	3.38	1.11
$v_{y}$	1.71	14.2	5.97
$v_{z}$	1.74	3.95	2.22
Velocity	2.69	15.4	6.56

**Table 4:** Average Processing Time for a Single Timestep.

	STC-PF	SCKF	SCKF	STC-PF
	+	+	+	+
	Single	Bilinear	GP	Parallel
t(s)	43.5	1.18	1.33	14.2

## Data Availability

SRTM data was obtained from USGS and are available at https://earthexplorer.usgs.gov/ with the permission of USGS. Detection data is not publicly available because this study was carried out as a defense-related project.

## Abbreviations

The following abbreviations are used in this manuscript:

DTED	Digital Terrain Elevation Data
PF	Particle Filter
SIR-PF	Sequential Importance Resampling Particle Filter
GP	Gaussian Process
LLA	Latitude, Longitude, and Altitude

## Appendix A. Derivative of Gaussian Process

A Gaussian process is a collection of random variables, any finite number of which have a joint Gaussian distribution. A Gaussian process is completely specified by its mean function and covariance function. The mean function and covariance function are defined as

(A1) $$\begin{array}{cc} m(x) & =E\left[f(x)\right],\\ k(x,x^{\prime }) & =E\left[\left(f(x)-m(x)\right)\left(f\left(x^{\prime }\right)-m\left(x^{\prime }\right)\right)\right], \end{array}$$

respectively [40]. Following the most common choice, zero mean function and squared exponential kernel are used throughout this paper. Namely,

(A2) m(x)=0,k(xm,xn)=α|exp−12∥xm−xn∥Γ2,

where ${∥x∥}_{\Gamma }=\sqrt{x^{T}\Gamma x}$. In most practical applications, we do not have direct access to function values themselves, only noisy versions thereof:

(A3) y=f(x)+ϵ,

where ϵ∼N(0,σ). Let *X* and *Y* be the concatenation of all observation points and corresponding measurements, respectively. Given the observation set (X,Y), the predictive distribution of the function value $f^{*}$ at arbitrary test points $X^{*}$ can be derived. Starting from the joint distribution of the observation set (X,Y) and the test set $\left(X^{*},f^{*}\right)$,

(A4) $$\left[\begin{array}{c} Y\\ f^{*} \end{array}\right]∼N\left(0,\left[\begin{array}{cc} k\left(X,X\right)+\sigma^{2}I & k(X,X^{*})\\ k(X^{*},X) & k(X^{*},X^{*}) \end{array}\right]\right).$$

Using basic arithmetic operations, Equation (A4) can be transformed into the following predictive distribution [40]:

(A5) $$\begin{array}{c} f^{*}|X^{*},X,Y∼N\left(\mu ,\Sigma \right),\\ \mathrm{where}\\ \mu =k{\left(X,X^{*}\right)}^{T}\cdot {\left(k\left(X,X\right)+\sigma^{2}I\right)}^{-1}\cdot Y,\\ \Sigma =k\left(X^{*},X^{*}\right)-k{\left(X,X^{*}\right)}^{T}\cdot {\left(k\left(X,X\right)+\sigma^{2}I\right)}^{-1}\cdot k\left(X,X^{*}\right). \end{array}$$

Furthermore, consider the derivative of the given GP. As differentiation is a linear operator, the derivative of a Gaussian process remains a Gaussian process as long as the kernel function is differentiable [41]. To find the joint probability of the observation set (X,Y) and the derivative observation set $\left(X_{d},Y_{d}\right)$, covariance between the function value and the derivative value as well as covariance among the derivative values should be described. First, let the derivative of the underlying function be

(A6) $$f_{d}={\left[\begin{array}{ccc} \frac{\partial f(x)}{\partial x_{1}} & \ldots & \frac{\partial f(x)}{\partial x_{D}} \end{array}\right]}^{T},$$

where *D* is the dimension of *x*. Then, the explicit expressions of the new covariance functions are

(A7) $$\begin{array}{cc} \mathrm{cov}\left({\left(f_{d}^{m}\right)}_{i},f^{n}\right)\; & =\frac{\partial }{\partial x_{i}}\mathrm{cov}\left(f^{m},f^{n}\right)\\ \; & =-\alpha \gamma_{i}\left(x_{i}^{m}-x_{i}^{n}\right)\exp\left(-\frac{1}{2}{∥x^{m}-x^{n}∥}_{\Gamma }^{2}\right),\\ \mathrm{cov}\left({\left(f_{d}^{m}\right)}_{i},{\left(f_{d}^{n}\right)}_{j}\right)\; & =\frac{\partial^{2}}{\partial x_{i}\partial x_{j}}\mathrm{cov}\left(f^{m},f^{n}\right)\\ \; & =\alpha \gamma_{i}\left(\delta_{i,j}-\gamma_{j}\left(x_{i}^{m}-x_{i}^{n}\right)\left(x_{j}^{m}-x_{j}^{n}\right)\right)\exp\left(-\frac{1}{2}{∥x^{m}-x^{n}∥}_{\Gamma }^{2}\right). \end{array}$$

Using basic arithmetic operations, the above expression is reduced to vector form:

(A8) $$\begin{array}{cc} \mathrm{cov}\left(f_{d}^{m},f^{n}\right)\; & =-k\left(x_{d}^{m},x^{n}\right)\Gamma \left(x_{d}^{m}-x^{n}\right)\\ \; & =k_{dx}\left(x_{d}^{m},x^{n}\right)\in R^{D\times 1},\\ \mathrm{cov}\left(f_{d}^{m},f_{d}^{n}\right)\; & =k\left(x_{d}^{m},x_{d}^{n}\right)\left(\Gamma -\Gamma \left(x_{d}^{m}-x_{d}^{n}\right){\left(x_{d}^{m}-x_{d}^{n}\right)}^{T}\Gamma \right)\\ \; & =k_{dd}\left(x_{d}^{m},x_{d}^{n}\right)\in R^{D\times D}. \end{array}$$

Suppose there are *N* observation points and *M* derivative observation points,

(A9) $$\begin{array}{cc} X & ={\left[\begin{array}{cccc} x^{1} & x^{2} & \ldots & x^{N} \end{array}\right]}^{T},\\ Y & ={\left[\begin{array}{cccc} y^{1} & y^{2} & \ldots & y^{N} \end{array}\right]}^{T},\\ X_{d} & ={\left[\begin{array}{cccc} x_{d}^{1} & x_{d}^{2} & \ldots & x_{d}^{M} \end{array}\right]}^{T},\\ Y_{d} & ={\left[\begin{array}{cccc} {\left(y_{d}^{1}\right)}^{T} & {\left(y_{d}^{2}\right)}^{T} & \ldots & {\left(y_{d}^{M}\right)}^{T} \end{array}\right]}^{T}. \end{array}$$

Finally, the joint distribution of the observation set (X,Y) and the derivative observation set $\left(X_{d},Y_{d}\right)$ can be described, assuming noise-free observation for the simplicity of the notation,

(A10) $$\left[\begin{array}{c} Y_{d}\\ Y \end{array}\right]∼N\left(0,\tilde{K}\right)$$

where

(A11) $$\tilde{K}=\left[\begin{array}{cc} {\tilde{K}}_{dd} & {\tilde{K}}_{dx}\\ {\tilde{K}}_{xd} & {\tilde{K}}_{xx} \end{array}\right],$$

(A12) $${\tilde{K}}_{dd}=k_{dd}\left(X_{d},X_{d}\right)=\left[\begin{array}{ccc} k_{dd}\left(x_{d}^{1},x_{d}^{1}\right) & \ldots & k_{dd}\left(x_{d}^{1},x_{d}^{M}\right)\\ \vdots & \ddots & \vdots \\ k_{dd}\left(x_{d}^{M},x_{d}^{1}\right) & \ldots & k_{dd}\left(x_{d}^{M},x_{d}^{M}\right) \end{array}\right]\in R^{MD\times MD},$$

(A13) $${\tilde{K}}_{dx}=k_{dx}\left(X_{d},X\right)=\left[\begin{array}{ccc} k_{dx}\left(x_{d}^{1},x^{1}\right) & \ldots & k_{dx}\left(x_{d}^{1},x^{N}\right)\\ \vdots & \ddots & \vdots \\ k_{dx}\left(x_{d}^{M},x^{1}\right) & \ldots & k_{dx}\left(x_{d}^{M},x^{N}\right) \end{array}\right]\in R^{MD\times N},$$

(A14) $${\tilde{K}}_{xx}=K_{xx}\left(X,X\right)=\left[\begin{array}{ccc} k_{xx}\left(x^{1},x^{1}\right) & \ldots & k_{xx}\left(x^{1},x^{N}\right)\\ \vdots & \ddots & \vdots \\ k_{xx}\left(x^{N},x^{1}\right) & \ldots & k_{xx}\left(x^{N},x^{N}\right) \end{array}\right]\in R^{N\times N},$$

and ${\tilde{K}}_{xd}={\tilde{K}}_{dx}^{T}$. Similar to the argument in Equations (A4) and (A5), the joint predictive distribution of the function value $f^{*}$ and derivative $f_{d}^{*}$ at the test point $X^{*}$ and derivative test point $X_{d}^{*}$, given the observation set (X,Y) and derivative observation set $\left(X_{d},Y_{d}\right)$ is

(A15) $$\left[\begin{array}{c} f^{*}\\ f_{d}^{*} \end{array}\right]|X^{*},X_{d}^{*},X,Y,X_{d},Y_{d}∼N\left(\tilde{\mu },\tilde{\Sigma }\right),$$

where

(A16) $$\begin{array}{c} \tilde{\mu }={\hat{K}}_{0}{\tilde{K}}^{-1}\left[\begin{array}{c} Y_{d}\\ Y \end{array}\right],\\ \tilde{\Sigma }={\hat{K}}_{1}-{\hat{K}}_{0}{\tilde{K}}^{-1}{\hat{K}}_{0}^{T}, \end{array}$$

and

(A17) $$\begin{array}{c} {\hat{K}}_{0}=\left[\begin{array}{cc} k_{xd}\left(X^{*},X_{d}\right) & k_{xx}\left(X^{*},X\right)\\ k_{dd}\left(X_{d}^{*},X_{d}\right) & k_{dx}\left(X_{d}^{*},X\right) \end{array}\right],\\ {\hat{K}}_{1}=\left[\begin{array}{cc} k_{xx}\left(X^{*},X^{*}\right) & k_{xd}\left(X^{*},X_{d}^{*}\right)\\ k_{dx}\left(X_{d}^{*},X^{*}\right) & k_{dd}\left(X_{d}^{*},X_{d}^{*}\right) \end{array}\right]. \end{array}$$

Now, given the sequence of test points, function value and gradient value of each test point can be inferred together. Because Equations (A15)–(A17) give mean and covariance, the function value and the slope can be sampled easily as well. If only observations of the function values are available, then $X_{d}$ and $Y_{d}$ are empty vectors.

## Appendix B. Choice of Hyper Parameters of Gaussian Process

According to the DTED specifications (Department of Defense(2000), Performance Specification, DTED, MIL-PRF-89020B), linear vertical absolute height error is less than 16 m and circular absolute geolocation error is less than 20 m for 90% of the data. Even though the global verification of the data is a challenging task, extensive ground truth data (e.g., Kinematic GPS data, GCPs) has been collected for the verification [37]. Consequently, it is found that linear vertical absolute height error is 6.2 m and circular absolute geolocation error is 8.8 m for 90% of the data.

Interestingly, the height error distribution is approximately a Gaussian, within an order of magnitude. Therefore, we can estimate the standard deviation of the height error $\sigma_{\mathrm{DTED}}$ to be 3.77 (m), because a 90% confidence interval is ±1.65σ.

Other hyper parameters are chosen empirically. Γ is inverse squared of 1 arc second ($\approx 2.78\times 10^{-4}$ deg) which makes the exponent part of the kernel function non-dimensional. Finally, α is properly selected so that the predictive mean sufficiently converges to the ground truth terrain elevation.

## Appendix C. Multinomial Resampling

Multinomial resampling was proposed with the first particle filter [42,43]. The multinomial resampling algorithm used in the implementation is illustrated in Algorithm A1.

**Algorithm A1:** Multinomial Resampling Algorithm.
